# Apolar Bioactive Fraction of *Melipona scutellaris* Geopropolis on *Streptococcus mutans* Biofilm

**DOI:** 10.1155/2013/256287

**Published:** 2013-06-13

**Authors:** Marcos Guilherme da Cunha, Marcelo Franchin, Lívia Câmara de Carvalho Galvão, Bruno Bueno-Silva, Masaharu Ikegaki, Severino Matias de Alencar, Pedro Luiz Rosalen

**Affiliations:** ^1^Department of Physiological Sciences, Dentistry School of Piracicaba, State University of Campinas (UNICAMP), Avenida Limeira 901, 13414-903 Piracicaba, SP, Brazil; ^2^College of Pharmaceutical Sciences, Federal University of Alfenas, 37130-000 Alfenas, MG, Brazil; ^3^Department of Agri-Food Industry, Food, and Nutrition, “Luiz de Queiroz” College of Agriculture, University of São Paulo, Avenida Pádua Dias 11, Piracicaba, SP, Brazil

## Abstract

The aim of this study was to evaluate the influence of the bioactive nonpolar fraction of geopropolis on *Streptococcus mutans* biofilm. The ethanolic extract of *Melipona scutellaris* geopropolis was subjected to a liquid-liquid partition, thus obtaining the bioactive hexane fraction (HF) possessing antimicrobial activity. The effects of HF on *S. mutans* UA159 biofilms generated on saliva-coated hydroxyapatite discs were analyzed by inhibition of formation, killing assay, and glycolytic pH-drop assays. Furthermore, biofilms treated with vehicle control and HF were analyzed by scanning electron microscopy (SEM). HF at 250 **μ**g/mL and 400 **μ**g/mL caused 38% and 53% reduction in the biomass of biofilm, respectively, when compared to vehicle control (*P* < 0.05) subsequently observed at SEM images, and this reduction was noticed in the amounts of extracellular alkali-soluble glucans, intracellular iodophilic polysaccharides, and proteins. In addition, the *S. mutans* viability (killing assay) and acid production by glycolytic pH drop were not affected (*P* > 0.05). In conclusion, the bioactive HF of geopropolis was promising to control the *S. mutans* biofilm formation, without affecting the microbial population but interfering with its structure by reducing the biochemical content of biofilm matrix.

## 1. Introduction

Dental caries is a biofilm-related infectious disease and is still the most prevalent oral disease [1]. Dental decay results mainly from the interaction between microorganisms in the mouth, tooth surface, and dietary components of the host, especially fermentable carbohydrates [2]. Among the microorganisms present in the complex oral microbiota, *Streptococcus mutans* has generally been regarded as the major etiologic agent of dental caries due to its ability to initiate the pathogenic biofilm formation [3].

The key role of *S. mutans* in the origin and installation of decay is due to its physiological characteristics that allow it to metabolize fermentable substrates, leading to pathological conditions observed in the disease. One of the important virulence factors of this microorganism is the glucan production from sucrose by enzymes known as glucosyltransferases (GTFs). These polysaccharides, particularly the insoluble ones, are responsible for the extracellular adhesion of bacteria to the tooth surface in the initial stages of the onset of the disease. Furthermore, these polysaccharides are responsible for forming a complex biofilm matrix, providing stability to this microbial community, and providing resistance to certain antimicrobial agents, as they can be used as energy storage [4–6]. Another important virulence factor is the ability of *S. mutans* to produce acid from fermentable substrates and also to survive in a low pH environment [7]. Environmental low-pH is responsible for enamel demineralization by dissolving the hydroxyapatite crystals present on tooth surface, thus initiating the pathological process of caries [8].

Although some strategies have been used in dental caries control, like fluoride and chlorhexidine digluconate, many compounds have shown promising activity on the virulence factors of this microorganism, providing new alternatives in the treatment and prevention of this disease [9]. Among these new alternatives, natural products have great merit, since approximately 70% of new antimicrobials available between 1981 and 2002 derived from natural sources [10].

Propolis is a natural plant resin collected by bees, and many studies have described its wide range of pharmacological effects, including activity against virulence factors of *S. mutans* [11–15]. Furthermore, some bioactive compounds isolated from Brazilian honeybee propolis have shown inhibitory capabilities in the development of caries in *in vivo* models [16]. Most studies on the biological activity and chemical composition provide added economic value to various types of propolis produced by the *Apis mellifera* bee, while others remain without a detailed description of their chemical composition and pharmacological activity. Among the several kinds of propolis, geopropolis is a different type collected by native and threatened stingless bees, such as *Melipona scutellaris*. Geopropolis is a kind of propolis composed by resin, wax, and soil collected by bee and deposited inside the beehive. It is widely used in the Northeast Region of Brazil although it has no added value [17]. However, there are few studies on this type of propolis, regarding its biological and chemical properties, describing its anti-inflammatory, antinociceptive, and antimicrobial activities, and also presenting polyprenylated benzophenones as major compounds instead of flavonoids, commonly found in other types of propolis [18–20]. Therefore, further studies on its biological activity are needed to add value to this product and elucidate its potential as a source of new bioactive compounds. The interesting potential of geopropolis has been reported by our research group, especially its hexane fraction, against oral bacteria [20]. Thus, the aim of this study was to investigate the influence of the hexane fraction of ethanolic extract of geopropolis on *in vitro S. mutans* biofilm.

## 2. Material and Methods

### 2.1. Propolis Samples and Fractionation

Crude samples of geopropolis from *M. scutellaris *(native stingless bee) were obtained from the city of Entre Rios (11°57′ S, 38°05′ W), in the state of Bahia, Northeast Brazil. Samples were extracted using ethanol (1 : 7, w/v) and dried according to Franchin et al. [18]. The ethanolic extract of geopropolis (EEGP) was subjected to chemical fractionation by a liquid-liquid extraction, based on a polarity gradient [18]. The liquid-liquid fractionation of the EEGP was carried out using three organic solvents, following the sequence: hexane (1 : 1, v/v) → chloroform (1 : 1, v/v) → ethyl acetate (1 : 1, v/v). After concentration, each fraction was tested against *S. mutans*, and hexane fraction (HF) was selected for presenting the highest antimicrobial activity required to proceed with the bioguided fractionation [20, 21]. Before using it, the HF was reconstituted with absolute ethanol at 3.2% (w/v) concentrations based on minimal inhibitory concentration for *S. mutans*.

### 2.2. Biofilm Assays

**Figure 1:**
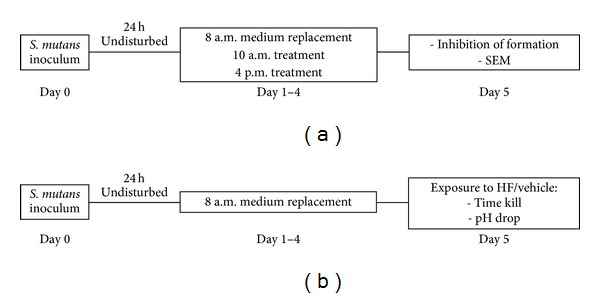
Experimental scheme of biofilm treatments. (a) Biofilms were treated with HF or vehicle control (ethanol 12.5%) twice daily after the initial 24 h, and then at the 5th day, the biofilms were analyzed by inhibition of formation experiment and by scanning electron microscopy (SEM). (b) Biofilms were grown with no treatment until the 5th day, and then the biofilms were exposed to HF or vehicle according to the experimental protocols of time kill and pH drop assays.

Biofilms of *S. mutans* UA159 (ATCC 700610, serotype *c*) were formed on saliva-coated hydroxyapatite (sHA) disks (Clarkson Chromatography Products, Inc., South Williamsport, PA; surface area 1.47 cm^2^). Human whole saliva was collected from one donor (Research Ethics Committee of the School of Dentistry of Piracicaba—University of Campinas—Protocol no. 047/2011), clarified by centrifugation (10000 g, 4°C, 10 min), sterilized and diluted (1 : 1) in adsorption buffer (AB—50 mM KCl, 1 mM KPO_4_, 1 mM CaCl_2_, 0.1 mM MgCl_2_, pH 6.5), and supplemented with the protease inhibitor phenylmethylsulfonyl-fluoride (PMSF) at a final concentration of 1 mmol/L. The sHA disks were placed in a vertical position, in 24-well plates, and inoculated with approximately 2 × 10^6^ CFU/mL in buffered ultrafiltered (10 kDa cutoff membrane; Prep/Scale; Millipore, MA) tryptone yeast extract (UFTYE, pH 7.0), with the addition of 1% (w/v) sucrose, at 37°C, 5% CO_2_. The biofilms were initially grown undisturbed during 24 hours, and then the culture medium was replaced daily during the 5 days of each experiment (total 115 h), according to Koo et al. [22]. The influence of the treatments with HF or vehicle control (ethanol 12.5%) on biofilm was analyzed according to the experimental scheme shown in Figure 1.

### 2.3. Inhibition of Biofilm Formation

To assess the effect of hexane fraction of geopropolis on the *S. mutans *biofilm formation, 24 h-old biofilms were treated twice daily (10 a.m. and 4 p.m., total of eight treatments, 1 min exposure each) with hexane fraction of geopropolis (250 and 400 *μ*g/mL) or vehicle control (ethanol 12.5%, v/v); both diluted in sterile AB (Figure 1(a)). The biofilms were washed five times in sterile saline 0.9% NaCl (to remove nonadhered cells), exposed during one minute to the agents, double washed in sterile saline 0.9% (to eliminate carry over effect), and finally returned to the culture medium. At the end of the experimental period (115 h) for biochemical collection data, the biofilms were removed and were subjected to ultrasound bath, sonication (30 s pulse; output 7 W), to provide the maximum recoverable viable counts. The homogenized suspension was analyzed for biomass (dry weight), bacterial viability (colony forming units CFU/mL), polysaccharide, and protein content. The extracellular water soluble polysaccharides (WSP), alkali-soluble polysaccharides (IP), and intracellular iodophilic polysaccharides (IPS) were extracted and quantified by colorimetric assays as detailed by Koo et al. [22] and Duarte et al. [23]; the exopolysaccharides were quantified by the phenolsulfuric method using glucose as standard [24], whereas IPS was quantified using 0.2% I2/2% KI solution and glycogen as standard, as described by DiPersio et al. [25]. The total protein was determined by colorimetric assays as detailed by Smith et al. [26].

### 2.4. Scanning Electron Microscopy (SEM)

For SEM analysis, the 115 h-old biofilm (treated as described in the inhibition of biofilm formation, Figure 1(a)) were washed in sterile NaCl 0.9% and then fixed with a 4% glutaraldehyde (v/v, in (phosphate buffered saline PBS, pH 7.4)) solution for 24 h. After that, biofilms were dehydrated in graded ethanol series (50, 70, 90, and 100%), dried for 24 h, and sputter coated with gold-palladium. The samples were then analyzed by SEM (JSM 5600LV, JEOL Tokyo, Japan) at 7000x [27].

### 2.5. Killing Assay

For the killing assay, 115 h-old biofilms (without treatment, Figure 1(b)) were exposed to HF at 250 and 400 *μ*g/mL, vehicle (ethanol 12.5%), and positive control (chlorhexidine digluconate 0.12%, Sigma-Adrich). At specific times (30, 60, 90, and 120 min) after exposure, biofilms were removed by ultrasound bath, sonication (30 s pulse; output 7 W), and then the homogenized suspension was serially diluted and plated on Brain Heart Agar. Plates were incubated in 5% CO_2_, at 37°C, for 48 h, and the number of colonies was determined (CFU/mL). Killing curves were constructed by plotting log 10 CFU/mL versus time over 120 min [16].

### 2.6. Glycolytic pH Drop

The effect of test agents on the acid production by *S. mutans *biofilms was evaluated using a technique described by Belli et al. [28] with some modifications. The 115 h-old biofilms grown in sHA disks (without daily treatments, Figure 1(b)) were washed in salt solution (50 mM KCl, 1 mM MgCl_2_·6H_2_O, pH 7.0) and exposed to HF (250 and 400 *μ*g/mL) or vehicle control (12.5% ethanol) for 90 min. The pHs of these solutions were adjusted to 7.2 with 0.1 M KOH solution, and then glucose was added (final concentration 1%, w/v). The decrease in pH was monitored with an Orion pH glass electrode attached to an Orion 290 A^+^ pHmeter.

### 2.7. Statistical Analysis

All assays were performed in duplicate of three independent experiments (*n* = 6). The data were subjected to the Shapiro-Wilk test followed by ANOVA and the Tukey-Kramer test to adjust for multiple comparisons, using the Biostat software version 5.0 for statistical visualization. The significance level was set at 5%.

## 3. Results

Table 1 shows the influence of bioactive HF of geopropolis on *S. mutans* biofilm formation on saliva-coated hydroxyapatite surface. The HF was not able to reduce the recoverable viable cells when compared to the vehicle control (*P* > 0.05). However, it significantly reduced the formation and accumulation of *S. mutans *biomass (*P* < 0.05) when compared with the ones treated with vehicle. Treatment with HF at 250 *μ*g/mL reduced 38% of biomass (dry weight) when compared to vehicle treatment, whereas the treatment with HF at 400 *μ*g/mL promoted a reduction of 53%. The biofilms treated with HF exhibited approximately 50–80% less polysaccharide than those treated with the vehicle control (*P* < 0.05). Except for the water soluble polysaccharide (WSP), the other kinds of glucans analyzed in this study had a significantly lower value than the vehicle control in biofilms treated with HF at both concentrations. Furthermore, the treatments also reduced significantly the total amount of protein in the biofilm, when compared to the vehicle control (*P* < 0.05).

**Figure 2:**
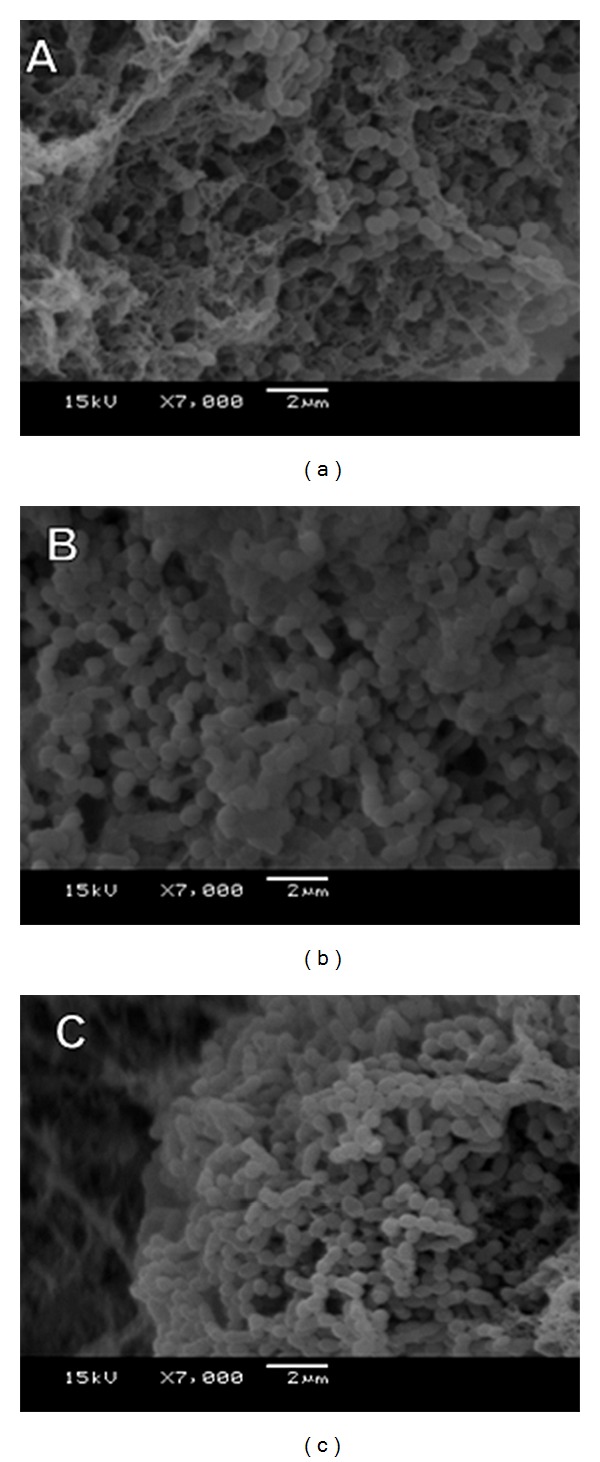
Effect of hexane fraction of geopropolis on the biofilm of *S. mutans*. (a) Vehicle control, (b) 250 *μ*g/mL, and (c) 400 *μ*g/mL.

SEM images (Figure 2) show the effect of HF at 250 and 400 *μ*g/mL on the biofilm accumulation. The images indicate a reduction of biomass accumulation by reducing the extracellular matrix without affecting the *S. mutans* growth.

**Figure 3:**
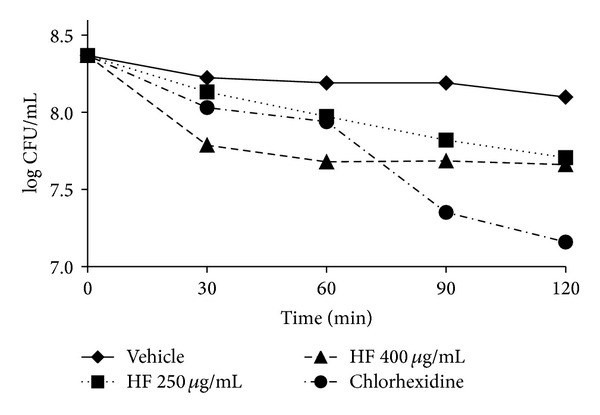
Time-kill curves of the hexane fraction on *S. mutans* biofilm.

**Figure 4:**
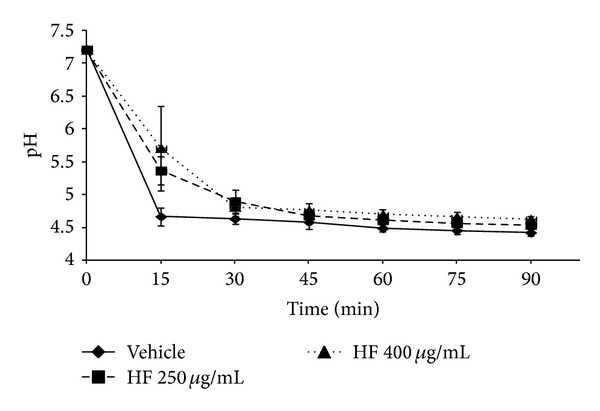
Influence of the hexane fraction and vehicle on glycolytic pH-drop in *S. mutans *biofilm.

Figure 3 shows that HF, at 250 *μ*g/mL and 400 *μ*g/mL, was able to reduce the cell viability of *S. mutans* on 115 h-old biofilm, but not significantly (*P* > 0.05). Furthermore, the acid production of *S. mutans *was not affected by HF at both concentrations (*P* > 0.05) (Figure 4), in the same condition (Figure 1(b)) once the treatments showed no difference from vehicle control. Thus, the HF was unable to interfere in the glycolytic production of acid by *S. mutans* biofilm which means that after the addition of glucose the HF did not prevent the drop in the curve of pH/acid production of the biofilm (Figure 4).

## 4. Discussion

Dental caries is a multifactorial disease, and its origin is associated to certain bacteria, especially *S. mutans*, which is responsible for initiating the cariogenic biofilm [1]. Thus, some alternative strategies to combat decay are focused on the control of the biofilm formed by this organism, acting on its virulence factors such as acidogenicity and polysaccharide formation [9]. Natural products have proved to be an important source of compounds that can act on these targets. Among these products, propolis has been noted for its known action on *S. mutans*, but geopropolis, a different kind of propolis, had not been studied yet on its ability to control the virulence factors of this microorganism. The aim of this study was to evaluate the activity of the hexane fraction from the ethanolic extract of *M. scutellaris* (stingless bee) geopropolis on *in vitro S. mutans* UA159 biofilm (ATCC 700610) and some of its virulence factors. Liberio et al. [29] recently studied the *Melipona fasciculata* geopropolis of the State of Maranhão (Northeast Brazil) from different biomes; however, their study differs from ours because its focus is on the viability of *S. mutans* ATCC 25175.

Geopropolis from *M. scutellaris* is active against *S. mutans* UA159 grown in planktonic state, and the nonpolar fraction (hexane) was selected because it has the highest activity among all fractions tested. In addition, the ethanolic extract of geopropolis and its HF significantly reduced cell adherence on *in vitro* biofilm [20]. In this study, HF of ethanolic extract of geopropolis was able to reduce the biomass (dry weight) compared to vehicle control (*P* < 0.05) when treated twice daily (total of eight treatments). We observed no decrease in the number of viable cells of *S. mutans*, suggesting a possible action on the biofilm matrix produced by this microorganism. However, the analyses of the biochemical composition of the biofilm indicated a significant reduction in the amounts of polysaccharides and protein. The interference on the matrix formation by HF led to a structural shift in the matrix, as these biochemical compounds are responsible for a three-dimensional conformation of the biofilm [30]. Confirming the action on the matrix, SEM images (Figure 2) showed a qualitative change in the structure and organization of the biofilm, as well as a loss of surface homogeneity. The apparent loss of homogeneity could be due to a simple superficial rearrangement, but the biochemical content data corroborate the hypothesis that HF promoted a lower accumulation of glucans and proteins in the matrix, when compared to the vehicle control. 

As HF was able to significantly reduce the amounts of proteins and extracellular polysaccharides, this agent showed an important impact on the accumulation and development of cariogenic biofilm. These polysaccharides, particularly insoluble extracellular (alkali-soluble) ones, may represent more than 40% of the biofilm dry weight, and they are considered responsible for promoting the binding and accumulation of microorganisms on the apatitic surface and to each other [30, 31]. Interfering in the synthesis of these compounds, HF would be useful in attenuating the mechanically stable structure formation of the biofilm, which protects the bacteria from environmental influences [32], including antimicrobial agents [4]. With these protections affected, the bacteria would be more susceptible to the host's defenses, making the attachment to tooth surface difficult and consequently the establishment of pathogenic biofilms. Furthermore, a change in extracellular glucan content of the biofilm caused by HF could influence the bacteria surface adherence, which would explain the findings of da Cunha et al. [20] on bacterial cell adhesion. 

Furthermore, a reduction in the amount of IPS was observed in biofilms treated with HF. These glucans are glycogen-like storage polymers with *α* 1–4 and *α* 1–6 linkages that can be fermented by bacteria under conditions in which exogenous carbohydrates are absent. The use of these polysaccharides by *S. mutans* leads to acid production that contributes to tooth demineralization [6]. This way, the long time exposure to HF could attenuate, in part, the pathogenic effects caused by the cariogenic biofilm.

This reduction on polysaccharide accumulation can be mainly due to a specific inhibitory activity on bacterial GTF or by affecting the expression of *gtf* genes. Among all polysaccharides analyzed, only the insoluble ones, ASP and IPS, were significantly inhibited, meanwhile the production WSP was not affected. The reduction of insoluble polysaccharides can be explained by a specific enzymatic inhibition of GTF B and C that produces glucans rich in *α* 1–3 and *α* 1–6 linkages, respectively. There was probably no effect on GTF D, which produces mainly soluble glucans, like WSP. 

Besides the analysis of action on biofilm matrix, HF was evaluated on cellular viability and acid production of *S. mutans*. At the concentrations tested in this study, HF was not able to affect the *S. mutans* viability and also had no interference on glycolytic acid production by the microorganism since this organism is acidogenic. Both results support the hypothesis of a selective activity of HF on the production of glucans by *S. mutans*, since HFs at these concentrations were not lethal to the microorganism and had no action over acid production, other important virulence factor of *S. mutans* that leads to superficial demineralization of the dental enamel by the dissolution of hydroxyapatite. Duarte et al. [16] showed a similar result for Brazilian propolis type 6, collected by *A. mellifera* bee. Although this type (6) of propolis has affected the acid production of bacteria, it did not affect the *S. mutans *UA159 viability, as well as HF in the present study. Moreover, in another report, Brazilian propolis type 6 was able to inhibit the *S. mutans* growth and adherence besides reducing the GTF activity in solution and adsorbed on sHA surface. Thereby, it would lead to a decrease in extracellular polysaccharide production and probably to a reduction on cell adherence and biofilm biomass, as observed for HF [12].

Such similar results between *M. scutellaris* geopropolis and Brazilian propolis type 6 could be explained in part by the same region of collection of these two varieties of propolis, as the biome of the collection determines the chemical content of propolis [33]. Even if collected by different bees, both products are obtained from the same region of the Atlantic Forest of the state of Bahia, Northeast Brazil, and according to preliminary studies from our research group they appear to have similar chemical profile, as geopropolis essentially has nonpolar compounds, like polyprenylated benzophenones, with the absence of flavonoids, as well as described for the Brazilian propolis type 6, that has hyperibone A as the main bioactive compound [14, 20]. 

Liberio et al. [29] evaluated the activity of the hydroalcoholic extract of geopropolis from *M. fasciculata* on oral pathogens. Their results indicated that this extract at 25 mg/mL is capable of reducing the viability of *S. mutans* (ATCC 25175) on biofilm formed in cell-culture plates. The different results observed when compared to the present study can be due to the distinct bacterial strains tested, once *S. mutans* ATCC 25175 is recognized as more resistant to antimicrobials, probably due to a differentiated and accelerated polysaccharide production in the extracellular matrix [34, 35]. Besides this, the active concentration used (greater than 60 times our highest concentration), the biofilm model (24-well polystyrene cell-culture plates), and mainly the biome where these geopropolis were collected (lakes, and babassu palm forests) can explain such different results. Our study reports the activity of *M. scutellaris* geopropolis, collected in the Atlantic forest, while *M. fasciculata* geopropolis originated from an ecosystem composed of mangroves, wetlands, lakes and babassu palm forests, which probably provides a different source of plant resins that can alter the geopropolis chemical composition. Furthermore, previous analyses have shown that *M. scutellaris* geopropolis has no flavonoids, while the activity of *M. fasciculata* geopropolis has been assigned to the presence of these compounds [20].

Finally, concerning new approaches in antimicrobial agents, Koo and Jeon [9] describe that natural products that act on the virulence factors of *S. mutans* without necessarily killing the bacteria have attracted attention as important sources of new effective drugs against dental caries. In the light of this view, our data show that geopropolis, especially its nonpolar fraction (hexane fraction), is a promising source of new compounds like polyprenylated benzophenones, capable to act on dental caries. Further studies are necessary to confirm such activity and to isolate/identify the active compounds. 

## 5. Conclusion

In summary, the hexane fraction of *M. scutellaris* (stingless bee) geopropolis from the Atlantic forest of the state of Bahia (Northeast Brazil) affected the biofilm formation by reducing the biomass, the polysaccharides, and protein content of biofilm matrix. However, it had no effect on the viability and acid production of *S. mutans*. This fraction is a natural source of promising bioactive substances to control the formation of *S. mutans* oral biofilm, without affecting the microbial population but interfering with its structure by reducing the biochemical content of biofilm matrix. 

Although further studies are required to identify the active compounds and their molecular mechanism of action, these compounds in HF may also be comprised in new formulations for clinical trials for oral plaque control, in order to prevent dental caries and oral biofilm related disease.

## Figures and Tables

**Table 1:** Effect of geopropolis fraction on the biochemical composition and viability of biofilm of *S. mutans. *

Treatment	Mean (±SD)					
	DW	ASP	IPS	WSP	Protein	BV
	(mg)	(*µ*g)	(*µ*g)	(*µ*g)	(mg)	(log CFU/mL)
Vehicle (Ethanol 12.5%)	4.63 (±0.59)	820.0 (±82.4)	289.9 (±44.9)	94.9 (±31.2)	1.14 (±0.20)	7.44 (±0.20)
HF 250 *μ*g/mL	2.83 (±0.88)*	403.3 (±70.0)*	73.6 (±19.6)*	75.3 (±25.2)	0.50 (±0.12)*	7.56 (±0.16)
HF 400 *μ*g/mL	2.18 (±0.28)*	352.2 (±62.1)*	52.4 (±05.7)*	72.2 (±31.3)	0.48 (±0.06)*	7.62 (±0.27)

*P < 0.05 when compared to vehicle control (ANOVA, Tukey-Kramer). DW: dry weight; ASP: alkali-soluble polysaccharide; IPS: intracellular iodophilic polysaccharide; WSP: water soluble polysaccharide; BV: bacterial viability.
